# Health and Budget Impact of Liquid-Biopsy-Based Comprehensive Genomic Profile (CGP) Testing in Tissue-Limited Advanced Non-Small Cell Lung Cancer (aNSCLC) Patients

**DOI:** 10.3390/curroncol28060441

**Published:** 2021-12-11

**Authors:** Yuti P. Patel, Donald Husereau, Natasha B. Leighl, Barbara Melosky, Julian Nam

**Affiliations:** 1 Hoffmann-La Roche Ltd., Mississauga, ON L5N 5M8, Canada; 2 School of Epidemiology, Public Health and Preventive Medicine, University of Ottawa, Ottawa, ON K1G 5Z3, Canada; don.husereau@gmail.com; 3 Princess Margaret Cancer Centre, Toronto, ON M5G 2C1, Canada; Natasha.Leighl@uhn.ca; 4 British Columbia Cancer Agency, Vancouver, BC V5Z 4E6, Canada; BMelosky@bccancer.bc.ca; 5 Hoffmann-La Roche Ltd., Grenzacherstrasse 124, Bldg 1/Floor 12, CH-4070 Basel, Switzerland; julian.nam@roche.com

**Keywords:** liquid biopsy, comprehensive genomic profiling, tissue-limited, health and budget impact, non-small cell lung cancer, next generation sequencing, single-gene testing, Canadian public payers

## Abstract

BACKGROUND AND OBJECTIVES: Molecular genetic testing using tissue biopsies can be challenging for patients due to unfavorable tumor sites, the invasive nature of a tissue biopsy, and the added time of booking a repeat biopsy (re-biopsy). Centers in Canada have found insufficient tissue rates to be approximately 10%, and even among successful biopsies, insufficient DNA in tissue samples is approximately 16%, triggering the lengthy process of re-biopsies. Using aNSCLC as an example, this study sought to characterize the health and budget impact of alternative liquid-biopsy(LBx)-based comprehensive genomic profile (CGP) testing in tissue-limited patients (TL-LBx-CGP) from a Canadian publicly funded healthcare perspective. MATERIAL AND METHODS: An economic model was developed to estimate the incremental cost and life-years gained as a population associated with adopting TL-LBx-CGP. The eligible patient population was modeled using a top-down epidemiological approach based on the published literature and expert clinician input. Treatment allocation was modeled based on biomarker prevalence in the published literature, and the availability of funded therapies. Costs included molecular testing, as well as drug, administrative, and supportive costs, and relevant health data included median overall survival and median progression-free survival data. RESULTS: Incorporation of TL-LBx-CGP demonstrated an overall impact of $14.7 million with 168 life-years gained to the Canadian publicly funded healthcare system in the 3-year time horizon.

## 1. Introduction

Cancer diagnosis and treatment are rapidly evolving, driven by enhanced understanding of cancer at the molecular level [1]. Over the past decade, personalized, targeted treatments based on the genomic features of a patient’s cancer are associated with improved response rates, progression-free survival (PFS), and overall survival (OS) compared to non-targeted, systemic treatments [2,3,4,5,6]. This is especially true for lung cancer, and in particular, for non-small cell lung cancer (NSCLC), which accounts for 85% of all lung cancers, with 63% of cases detected in advanced stages (aNSCLC) [7]. Given that NSCLC is complex and driven by a broad range of potentially targetable genetic alterations, deploying a robust approach to molecular genetic testing is increasingly essential to support informed decisions on optimal therapies for individual patients.

Currently, in Canada, actionable mutations which guide treatment selection for hlNSCLC are limited to anaplastic lymphoma kinase 1 (ALK1), epidermal growth factor receptor (EGFR), roto-oncogene tyrosine-protein kinase 1 (ROS1), and B-Raf proto-oncogene (BRAF) [8,9,10,11]. However, genetic testing for these alterations is typically performed through single-gene and hotspot multi-gene testing methods which are unable to detect copy number variations or rearrangements which can aid in clinical interpretation [12]. Furthermore, tissue exhaustion can limit the number of single tests that can be performed [12]. These challenges may be mitigated with the use of comprehensive genetic profiling (CGP) using massive parallel, or next-generation sequencing (NGS) technologies to more broadly test for genomic alterations with a single test. NGS allows for the identification of low frequency mutations missed through conventional panels, more precise diagnosis and prognosis prediction, the selection of patients for optimal targeted therapies, and the ability to identify resistance mutations to guide subsequent lines of therapy [13]. The extensive capabilities of NGS now offer oncologists the option to deliver a higher level of personalized care to cancer patients that optimizes treatment choices and results in improved outcomes [13]. In the therapeutic area of hematological cancer, studies have demonstrated the application of liquid-biopsy-based biomarkers for the diagnosis of solid and liquid cancers [14].

Testing is often complicated for aNSCLC patients due to issues including unfavorable tumor site, clinical frailty of lung tissue, lack of tissue obtained after a biopsy, the invasive nature of a tissue biopsy, and the time and availability of resources for performing a biopsy [15]. Centers in Canada have found insufficient tissue rates to be approximately 10%, and even among successful biopsies, insufficient DNA in tissue samples occurs in 16% of tests, triggering a lengthy process of repeat biopsies (re-biopsies) [16]. Moreover, of patients with adequate tissue for molecular genetic testing, 75% are tested using a single gene methodology, but approximately 29% cannot continue with additional genetic testing due to tissue exhaustion [17,18]. In cases in which appropriate testing is not employed, targeted treatment is unavailable to a number of NSCLC patients, and the ability to identify patients for some clinical trials is limited. This restricts physicians to systemic chemotherapies which could result in worse clinical outcomes than if they could match to an appropriate, targeted therapy. This highlights a gap in the treatment journey amongst “tissue-limited” (TL) aNSCLC patients, defined as individuals who encounter the following: insufficient tissue for molecular genetic testing; insufficient DNA in tissue samples requiring re-biopsies; or exhausted tissue from single-gene testing. 

Circulating tumor DNA (ctDNA), commonly referred to as liquid biopsy (LBx), presents a case for alternative molecular genetic profiling in these tissue-limited (i.e., TL-LBx) scenarios. Clinical validation studies have demonstrated that LBx-based NGS shows a high positive concordance to tissue-based NGS [19]. An example of LBx-based CGP testing is FoundationOne Liquid^®^ CDx, launched in June 2020. Using a blood sample, FoundationOne Liquid^®^ analyzes and detects novel and known variants of the four main classes of genomic alterations in more than 300 genes and genomic signatures, such as blood tumor mutational burden (bTMB), and microsatellite instability (MSI) status, to help inform treatment decisions and identify potential clinical trials for patients with advanced cancer [20]. However, despite the availability of advanced testing technologies, the utilization of LBx-CGP remains low. 

Though there appears to be a clear benefit to using a TL-LBx-CGP approach, policymakers still need to understand the impact on patients and healthcare expenditures to facilitate its widespread adoption. To address this, and using aNSCLC as an example, this study sought to characterize the health and budget impact of alternative TL-LBx-CGP testing in tissue-limited patients from a Canadian publicly funded healthcare perspective.

## 2. Materials and Methods

### 2.1. Model Structure

**Figure 1:**
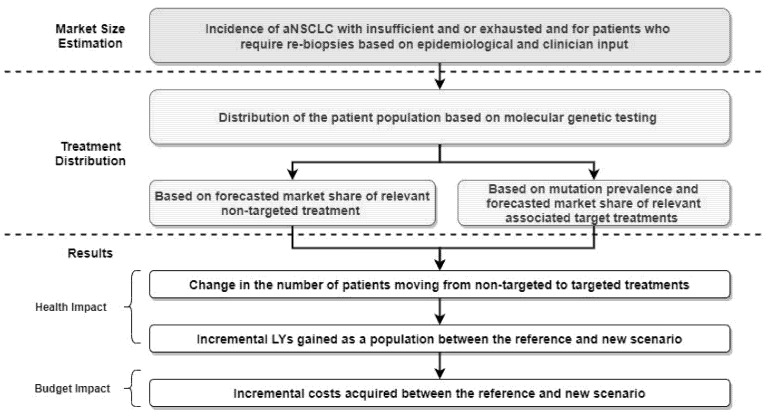
Health and budget impact model overview.

The health and budget impact of adopting alternative TL-LBx-CGP in aNSCLC patients was assessed from a Canadian publicly funded healthcare perspective over a three-year time horizon. An economic model was developed in a Microsoft Excel spreadsheet which estimated the number of patients who could receive targeted therapies rather than non-targeted therapies due to identification of an actionable mutation, and then estimated the incremental population life-years (LYs), and costs associated with funding TL-LBx-CGP (refer to Figure 1). The eligible patient population was modelled using a reference (TL-LBx-CGP, not publicly funded) and new (TL-LBx-CGP, publicly funded) scenario through a two-step process: market size estimation and treatment distribution.

#### 2.1.1. Market Size Estimation

**Figure 2:**
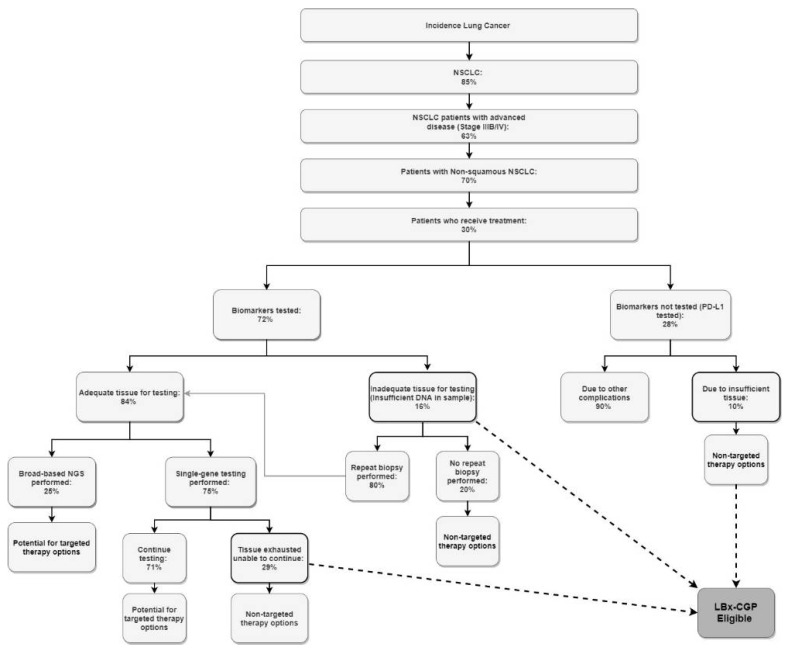
Market size estimation: patient testing journey.

To estimate market size, a top-down epidemiological approach based on the published literature and expert clinician input was used (Figure 2). Canadian incident lung cancer was used as a starting point, and subsequent epidemiological filters were applied until the biomarker testing point in the patient journey. Of the patients who receive treatment, 28% of aNSCLC patients don’t receive biomarker testing, in which 10% are due to insufficient tissue [13]. Of the 72% that receive biomarker testing, 16% require re-biopsies due to inadequate DNA in the tissue sample [13]. For those patients with adequate tissue samples, 25% receive multigene assay panel testing, whereas 75% have single-gene testing, as depicted in Figure 2 and Table 1 and Table 2 [15,16,17]. As depicted in Table 2, the adoption of TL-LBx-CGP leads to all patients having access to molecular genetic testing (versus the reference scenario) through either: NGS; single-gene testing; or TL-LBx-CGP; and no patients in the “no testing” category.

#### 2.1.2. Treatment Distribution

**Figure 3:**
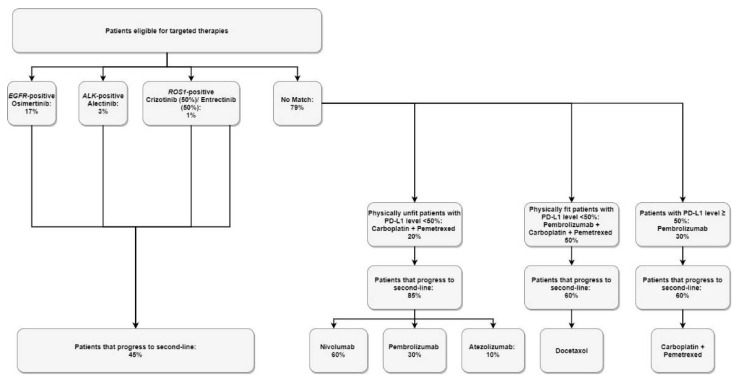
Treatment distribution: targeted therapies.

**Figure 4:**
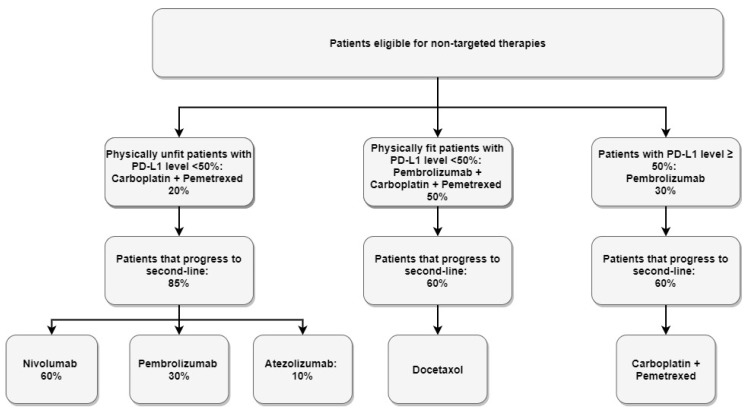
Treatment distribution: non-targeted therapies.

Eligible patients in each scenario receive publicly-funded first-line treatment options based on the likelihood of receiving molecular genetic testing results, and biomarker prevalence rates (Table 3). It was assumed that PD-L1 was tested for all patients. Biomarkers tested in the new scenario are based on the NCCN guidelines for mutations recommended for testing (EGFR, ALK, ROS1, BRAF V600E), as well as the NTRK mutation; however, currently in Canada, there no funded therapies for BRAF or NTRK positive, and thus, those patients were placed in the “no match” arm. The model assumed patients who received molecular genetic testing are eligible for targeted therapies (Figure 3), and patients who received no molecular genetic testing are eligible for non-targeted therapies (Figure 4). Upon progression from first-line treatment, patients are distributed appropriately into approved, publicly funded second-line treatments.

### 2.2. Health Impact Analysis

**Figure 5:**
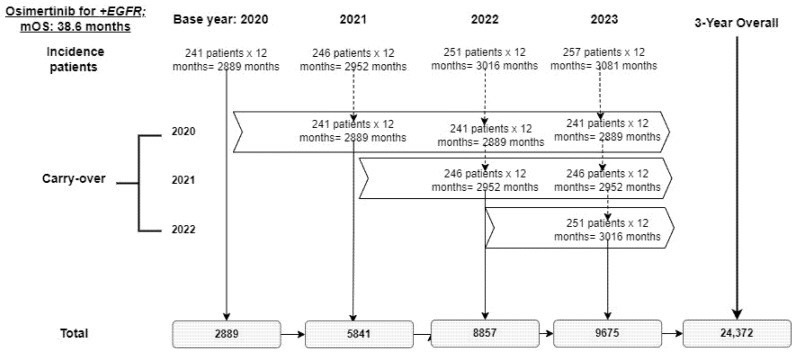
Health impact sample calculation.

The base case for the health impact was informed by the change in the number of patients moving from non-targeted to targeted therapies, and the associated incremental LYs gained for annual population cohorts over a 3-year time horizon. Change in the number of patients moving from non-targeted therapies to targeted was estimated by monitoring the incident patients distributed into the different types of therapies (chemotherapy, immunotherapy, chemo-immunotherapy, and targeted therapy) per year in the reference versus new scenario. The population LYs in each scenario were calculated by multiplying the number of patients distributed to the respective treatments by the median overall survival (mOS) (Table 4). Carry-over beyond one year was assigned to cohorts receiving treatments with mOS values greater than 12 months. As an exploratory result, the lifetime LYs gained as a population were calculated by carrying-forward all incident patients captured within the 3-year time horizon until the end of their mOS. A sample calculation for incremental population LYs using osimertinib is presented in Figure 5.

### 2.3. Budget Impact Analysis

#### 2.3.1. Cost Inputs

There were four cost categories included for molecular testing costs: NGS; single-gene testing; TL-LBx-CGP; and re-biopsies. Table 5 presents the cost per patient for each of the testing methodologies considered. To be conservative, the list price of FoundationOne Liquid CDx^®^ was utilized as a proxy for a commercial TL-LBx-based CGP test. An assumption in this analysis is that two negative single gene tests completed prior to tissue exhaustion is equivalent to twice the list price ($652) [8]. The cost of NGS, single gene, and re-biopsies includes all direct hospital costs and professional fees.

Drug costs included in each line of therapy were based on list-price of approved and publicly funded interventions. The total cost per treatment was determined by the Health-Canada-recommended dosage and unit cost (details outlined in the Supplementary Materials) [32]. The overall annual drug cost per patient was calculated according to the number of treatment cycles in a year, using median progression free survival (mPFS) data as a proxy for average treatment length (Table 4). 

Administrative costs included chair time [33,34], pharmacists [34], clinician consultation [35], and pre-medication cost [32] (details outlined in Supplementary Materials). For oral treatments, the total annual costs per patient were calculated on a weekly basis, and for IV treatments, the costs calculated per every 21 days (typically the length of one cycle). Supportive costs included clinician consultation [35], laboratory testing [36], and imaging procedure costs [31,35] (details outlined in Supplementary Materials). Similar to administrative costs, total annual oral treatments supportive costs per patient were calculated on a weekly basis, and for IV treatments, the costs are per every 21 days.

The total annual costs (aggregated and disaggregated) per patient per therapy are presented in Appendix A.

#### 2.3.2. Analysis and Assumptions

The net annual impact was estimated by determining the number of patients distributed to each of the different treatments, and calculating the treatment duration, in cycles. Median-progression-free (mPFS) survival data (Table 4) associated with each treatment was used as a proxy for treatment duration. Carryover costs for treatments with mPFS >12 months were handled similarly to LYs calculations with mOS >12 months. The total cost per treatment in each scenario was determined by multiplying the total number of cycles in a year by each of the cost categories. Further, total costs per treatment were multiplied by total number of patients distributed to the treatment, and summed in each scenario to calculate the annual incremental difference (new scenario less reference scenario) starting with the base year, 2020, followed by a 3-year time horizon.

### 2.4. Sensitivity and Scenario Analysis 

Uncertainty was explored through one-way sensitivity analyses (Table 6). The Canadian publicly funded healthcare system is composed of federated healthcare structures, is siloed, and cost categories are accounted for in various budgets. As such, a scenario from a Canadian testing budget perspective was performed, in which only molecular genetic testing costs were considered. 

## 3. Results

### 3.1. Health Impact 

**Figure 6:**
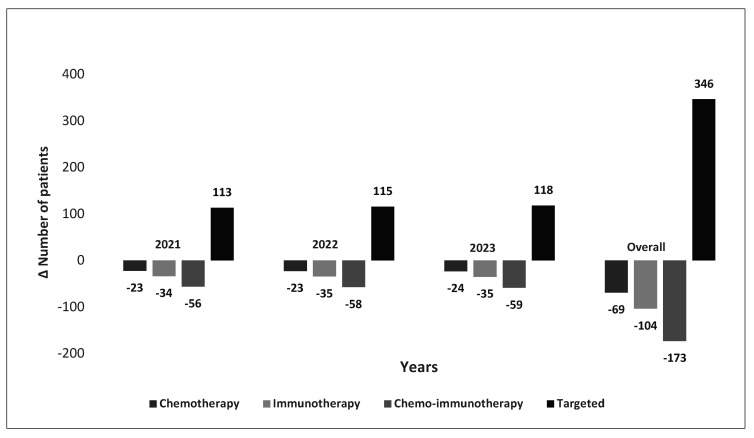
Change in the number of patients moving from non-targeted to targeted therapies.

Public funding of TL-LBx-CGP resulted in an estimated 346 more patients accessing targeted therapies over the 3-year time horizon as opposed to chemotherapies (78 fewer patients), immunotherapies (117 fewer patients), and chemo-immunotherapies (195 fewer patients) (Figure 6). Because of access to targeted therapies, the entire tissue-limited population gained 2 LYs in year 1, 34 LYs in year 2, 131 LYs in year 3, and a combined total of 168 LYs in the first 3 years (Table 7, Table 8 and Table 9). Five-hundred and seventy-four LYs were also accrued beyond the 3-year timeframe; however, additional downstream costs were not estimated for this period.

### 3.2. Budget Impact

For the base case, the impact to the Canadian publicly funded healthcare system resulted in an expenditure of −$1,415,057 in year 1, $7,028,238 in year 2, and $9,089,833 in year 3, for a total 3-year impact of $14,703,014 (Table 10).

### 3.3. Sensitivity and Scenario Analysis

#### 3.3.1. Sensitivity Analysis 

The one-way sensitivity analyses varied each assumption made in the development of the model by a specified amount. The net difference and percent change from base case of the most sensitive parameters is presented in Table 11. The estimated costs of testing were most sensitive to assumptions about broad-based NGS testing performed and the proportion of patients unable to continue due to tissue exhaustion. The full sensitivity analysis results are listed in Appendix B.

#### 3.3.2. Scenario Analysis

The net expenditure for the scenario analysis from a Canadian testing budget perspective was $3,854,907 in year 1, $3,938,368 in year 2, and $4,023,674 in year 3, for a total 3-year impact of $11,816,949 (Table 10).

## 4. Discussion

The reliance on tissue-based biomarker testing in lung cancer presents a barrier to accessing timely and optimal care for the tissue-limited patient population. Using aNSCLC as an example, this study presents a case to assess the health and budget implications of adopting LBx-based CGP testing in tissue-limited patients (insufficient tissue/exhausted tissue and who require re-biopsies) from a Canadian healthcare perspective (TL-LBx-CGP). 

Our findings suggest widespread public funding of TL-LBx-CGP could allow as many as 346 more aNSCLC patients to obtain targeted therapies as opposed to chemotherapies, immunotherapies, and chemo-immunotherapies (Figure 6). Consequently, an increased number of patients on targeted therapies translates to improved health outcomes, with the first 3 years of funding TL-LBx-CGP leading to an incremental gain of 168 LYs within Canadian publicly-funded healthcare systems. An additional 574 LYs are gained beyond the 3-year time horizon (Table 8 and Table 9); however, associated downstream costs were not estimated for this time period. The incremental gain in the new scenario versus the reference can be attributed directly to the increase in the number of patients receiving targeted therapy as opposed to systemic therapies. 

The incremental gain from the health impact was associated with an overall budget impact of approximately $14.7 M in the first 3 years of implementation (Table 10). This includes a reduction in costs in year 1 with both drug (−$3.8 M) and administrative costs (−$1.6 M). Cost reduction in year 1 is primarily driven by reducing the use of triplet chemo-immunotherapy (Carboplatin+Pemetrexed+Pembrolizumab), which is more costly compared to tyrosine kinase inhibitors (TKIs) therapy (Table 6). In the reference scenario, an increased number of tissue-limited patients were allocated to the triplet chemo-immunotherapy due to the lack of an appropriate biomarker test. 

Moving from year 1 to year 2, cost reduction is no longer observed as improved health outcomes associated with TKIs (in the new scenario) allow patients to remain on treatment longer without progression, increasing drug costs. Improved health outcomes through improved access to TKIs also translates to carry-over patients that further increase healthcare expenditure. Despite this, some cost offsets due to lowered administrative costs are achieved, as an increased number of patients on oral TKIs reduces the demand for chemotherapies, immunotherapies, and chemo-immunotherapies requiring intravenous administration. Lower administrative costs are persistent throughout the time horizon, as the number of patients on targeted therapies increases from year to year. 

A strength of this study is the holistic perspective applied, which not only highlights the testing journey of a patient, but also how testing influences treatment decisions, leading to the accumulation of appropriate costs and health outcomes. Despite recognizing the need to consider a wider impact to the health system, we are aware some Canadian budget holders may want to more narrowly focus on impact to the laboratory budget alone. As such, we also examined the impact of funding TL-LBx-CGP from the Canadian testing budget perspective in a scenario analysis. From this perspective, only molecular genetic testing costs were considered, resulting in a total 3-year impact of $11 M.

Despite the need to consider a system-wide impact, previous studies have tended to focus on studying testing or targeted therapies in a silo. A similar study by Johnston et al. focused on the economic and health impact of adopting NGS and TL-LBx-CGP in NSCLC, which resulted in a lower 3-year budget (~$4.5 M) than this study [37]. The Johnston et al. study focused mainly on tissue-biopsy, and made a simplifying assumption that 5% of patients had unavailable tissue for testing. This study adds a more detailed and disaggregated breakdown of tissue-limited aNSCLC patients. Additionally, this study includes all potential drug treatment costs.

A potential limitation of our analysis is that it relies heavily on epidemiological, molecular genetic testing and cost inputs. As such, assumptions made in the development of the model were tested through a series of one-way sensitivity analyses (Table 11). Notably, tissue exhaustion rates were one of the most influential parameters, in which a variance of 10% caused an 88% from the reference case. The lack of an accurate estimate is a limitation of our findings, as it necessarily added simplifying assumptions regarding best-practices surrounding tissue conservation during molecular genetic testing procedures. In practice, the average percentage of exhausted tissue may be lower, as laboratory professionals are motivated to preserve as much of a tissue sample as possible. 

Further, results in the model are distinctly sensitive to the adoption rates of NGS vs. single-gene testing, and an increase in NGS adoption (versus single-gene testing) presents a case for substantial cost-savings. Nonetheless, heterogeneity of testing adoption rates, treatment patterns, and unit costs across Canada can greatly impact the overall results. Also, this study did not evaluate the pharmacoeconomic impact of clinical trial options, which generally contribute to improved health outcomes (versus real-world practice), and are backed using funds outside the healthcare budget [38]. Future analysts should also consider that technology is likely to be more productively efficient over time as more therapies are authorized and reimbursed, as well as the inclusion of productivity measures to capture the societal impact associated with the adoption of this technology for TL-LBx-CGP. 

## 5. Conclusions

In summary, this study sought to characterize the health and budget impact of publicly funding LBx-CGP in tissue-limited aNSCLC patients from a Canadian healthcare perspective. The modelling resulted in 346 more patients accessing targeted therapies, adding 168 population LYs in the first 3 years of implementation (with the opportunity to gain 574 LYs beyond the time horizon). The added health impact was attributed to a modest budget impact of $14.7 M over 3 years.

## Figures and Tables

**Table 1:** (**a**) Market size estimation—reference scenario. (**b**) Market size estimation—new scenario.

**(a) Market Size Estimation—Reference Scenario**				
**Patient Flow**	**2020**	**2021**	**2022**	**2023**
Lung cancer patients	23,093	23,593	24,104	24,626
NSCLC patients	19,629	20,054	20,488	20,932
Locally advanced/metastatic	12,366	12,634	12,908	13,187
Non-squamous NSCLC	8657	8844	9035	9231
Patients who receive treatment	2597	2653	2711	2769
Biomarker testing not performed Patients with insufficient tissue for biomarker testing *	727	743	759	775
	73	74	76	78
Biomarker testing performed	1870	1910	1952	1994
Inadequate tissue for conventional testing	299	306	312	319
Re-biopsy performed	239	245	250	255
Re-biopsy not performed *	60	61	62	64
Adequate tissue for testing	1810	1849	1889	1930
Broad-based NGS testing performed *	452	462	472	483
Sequential single-gene testing performed *	1357	1387	1417	1448
Patient able to continue sequential testing	964	985	1006	1028
Patients unable to continue due to tissue exhaustion *	394	402	411	420
**Total number of patients**	**1943**	**1985**	**2028**	**2071**
**(b) Market Size Estimation—New Scenario**				
**Patient Flow**	**2020**	**2021**	**2022**	**2023**
Lung cancer patients	23,093	23,593	24,104	24,626
NSCLC patients	19,629	20,054	20,488	20,932
Locally advanced/metastatic	12,366	12,634	12,908	13,187
Non-squamous NSCLC	8657	8844	9035	9231
Patients who receive treatment	2597	2653	2711	2769
Biomarker testing not performed **TL-LBx-CGP Eligible:** Patients with insufficient tissue for biomarker testing *	727	743	759	775
	73	74	76	78
Biomarker testing performed	1870	1910	1952	1994
**TL-LBx-CGP Eligible:** Inadequate tissue for conventional testing	299	306	312	319
Re-biopsy performed	239	0	0	0
Re-biopsy not performed *	60	0	0	0
Adequate tissue for testing	1810	1605	1639	1675
Broad-based NGS testing performed *	452	401	410	419
Sequential single-gene testing performed *	1357	1203	1230	1256
Patient able to continue sequential testing	964	854	873	892
**TL-LBx-CGP Eligible:** Patients unable to continue due to tissue exhaustion *	394	349	357	364
**Total number of patients**	**1943**	**1985**	**2028**	**2071**

* These numbers were summed to determine the total number of patients (market size). Values in the table above are subject to rounding.

**Table 2:** (**a**) Molecular genetic testing distribution—reference scenario. (**b**) Molecular genetic testing distribution—new scenario.

**(a) Molecular Genetic Testing Distribution—Reference Scenario**				
**Molecular Genetic Test**	**2020**	**2021**	**2022**	**2023**
**Eligible Patients**	**1943**	**1985**	**2028**	**2071**
NGS				
NGS Only	393	401	410	419
NGS + Re-biopsy	60	61	62	64
Single-gene testing				
Single-gene testing only	836	854	873	892
single-gene testing + Re-biopsy	127	130	133	136
No testing	526	538	549	561
TL-LBx-CGP	0	0	0	0
**(b) Molecular Genetic Testing Distribution—New Scenario**				
**Molecular Genetic Test**	**2020**	**2021**	**2022**	**2023**
**Eligible Patients**	**1943**	**1985**	**2028**	**2071**
NGS				
NGS Only	393	401	410	419
NGS + Re-biopsy	60	0	0	0
Single-gene testing				
Single-gene testing only	836	854	873	892
single-gene testing + Re-biopsy	127	0	0	0
No testing	526	0	0	0
TL-LBx-CGP	0	729	745	761

Values in the table above are subject to rounding.

**Table 3:** (**a**) Treatment distribution—reference scenario. (**b**) Treatment distribution—new scenario.

**(a) Treatment Distribution—Reference Scenario**				
**2020**	**2020**	**2021**	**2022**	**2023**
**Eligible Patients**	**1943**	**1985**	**2028**	**2071**
*First-line Therapy*				
Osimertinib	241	246	251	257
Alectinib	42	43	44	45
Crizotinib	7	7	7	8
Entrectinib	7	7	7	8
Carboplatin Pemetrexed	329	336	343	351
Carboplatin + Pemetrexed + Pembrolizumab	823	840	859	877
Pembrolizumab	494	504	515	526
*Second-Line Therapy*				
Carboplatin + Pemetrexed	430	439	449	458
Docetaxel	494	673	687	702
Nivolumab	168	229	234	239
Pembrolizumab	84	114	117	119
Atezolizumab	28	38	39	40
Total number of patients progressed to 2L	1203	1493	1526	1559
**(b) Treatment Distribution—New Scenario**				
**Treatment**	**2020**	**2021**	**2022**	**2023**
**Eligible Patients**	**1943**	**1985**	**2028**	**2071**
*First-line Therapy*				
Osimertinib	241	337	345	352
Alectinib	42	60	61	62
Crizotinib	7	10	10	10
Entrectinib	7	10	10	10
Carboplatin Pemetrexed	329	314	320	327
Carboplatin + Pemetrexed + Pembrolizumab	823	784	801	818
Pembrolizumab	494	470	481	491
*Second-Line Therapy*				
Carboplatin + Pemetrexed	430	470	480	490
Docetaxel	494	470	481	491
Nivolumab	168	160	163	167
Pembrolizumab	84	80	82	83
Atezolizumab	28	27	27	28
Total number of patients progressed to 2L	1203	1207	1233	1259

**Table 4:** Health outcomes data.

Treatment	Mutation	mOS (Months)	mPFS (Months)	Source
Osimertinib	*EGFR*	38.6	18.9	[21]
Alectinib	*ALK*	58.4	34.8	[22]
Crizotinib	*ROS1*	51.4	19.3	[23]
Entrectinib	*ROS1*	62.3	15.7	[24]
Carboplatin + Pemetrexed (1L/2L)	Unknown levels of PD-L1	10.7	4.9	[25]
Pembrolizumab + Carboplatin + Pemetrexed	Negative/low levels PD-L1	22	9.0	[25]
Pembrolizumab (1L)	High levels of PD-L1	30	10.3	[26]
Docetaxel	N/A	4.2	2.0	[27]
Nivolumab	N/A	12.2	2.3	[28]
Pembrolizumab (2L)	N/A	10.5	3.8	[29]
Atezolizumab	N/A	13.8	2.8	[30]

1L: First-line treatment; 2L: Second-line treatment.

**Table 5:** Annual molecular genetic testing costs (per patient).

Cost Categories	Cost per Year
NGS	$1919.00 [12]
Single- gene testing (two tests)	$1304.00 [12]
TL-LBx CGP	$6193.60 [12]
Re-biopsies	$1948.26 [31]

**Table 6:** Health outcomes data.

Parameter	Base Case	Sensitivity Analysis
**Epidemiological Inputs**		
Lung cancer patients with NSCLC	85%	±10%
NSCLC patients with advanced disease (Stage 3B/4A)	63%	±10%
Patients with non-squamous NSCLC	70%	±10%
Patients who receive treatment	30%	±10%
**Molecular genetic testing inputs**		
Biomarker not tested, excluding PD-L1	28%	±10%
Patients with insufficient tissue for biomarker testing	10%	±5%
Adequate tissue for testing	84%	±10%
Re-biopsy performed	80%	±10%
Broad-based NGS testing performed	25%	±25% −50% +75%
Patients unable to continue due to tissue exhaustion	29%	±10%
**Molecular genetic testing cost inputs**		
TL-LBx-CGP (FoundationOne Liquid CDx^®^)	$6193.60	Discount: 25%, 50%, 75%
Single-gene testing	$1304.00	Discount: 25%, 50%, 75%
Re-biopsy	$1948.26	Discount: 25%, 50%, 75%
Broad-based NGS	$1919.00	Discount: 25%, 50%, 75%

**Table 7:** Population LYs gained—reference scenario.

Treatment	2020	2021	2022	2023	3-Year Combined	Life-Time
Osimertinib	2889	5841	8857	9675	24,372	35,511
Alectinib	510	1031	1563	2107	4700	9743
Crizotinib	85	172	260	351	783	1419
Entrectinib	85	172	260	351	783	1738
Carboplatin + Pemetrexed	3521	3597	3675	3754	11,025	11,025
Carboplatin + Pemetrexed + Pembrolizumab	9871	18,310	18,706	19,111	56,127	64,898
Pembrolizumab	5922	11,973	15,193	15,522	42,688	55,252
**Total LYs (months)**	**22,883**	**41,095**	**48,515**	**50,871**	**140,480**	**179,586**

Values in the table above are subject to rounding.

**Table 8:** Population LYs gained—new scenario.

Treatment	2020	2021	2022	2023	3-Year Combined	Life-Time
Osimertinib	2889	6938	11,074	13,036	31,048	46,325
Alectinib	510	1224	1954	2700	5878	12,630
Crizotinib	85	204	326	450	980	1842
Entrectinib	85	204	326	450	980	2251
Carboplatin + Pemetrexed	3521	3355	3428	3502	10,285	10,285
Carboplatin + Pemetrexed + Pembrolizumab	9871	17,632	17,450	17,827	52,909	61,092
Pembrolizumab	5922	11,566	14,372	14,480	40,418	52,138
**Total LYs (months)**	**22,883**	**41,124**	**48,928**	**52,445**	**142,498**	**186,562**

Values in the table above are subject to rounding.

**Table 9:** Incremental population LYs gained.

Scenario	Base Year: 2020	2021	2022	2023	3-Year Combined	Lifetime Combined
Reference	22,883	41,095	48,515	50,871	140,480	179,586
New	22,883	41,124	48,928	52,445	142,498	186,562
**Incremental LYs (months)**	**0**	**29**	**414**	**1574**	**2018**	**6976**
**Incremental LYs (years)**	**0**	**2**	**34**	**131**	**168**	**581**

Values in the table above are subject to rounding.

**Table 10:** Budget impact—base case and scenario analysis.

	Base Year: 2020	2021	2022	2023	3-Year Combined
Molecular Testing Cost	$0	−$3,787,555	$4,007,510	$5,881,902	$6,101,857
Drug Cost	$0	−$1,646,575	−$1,668,394	−$1,701,711	−$5,016,680
Administrative Cost	$0	$164,166	$750,755	$885,968	$1,800,888
Supportive Cost	$0	$3,854,907	$3,938,368	$4,023,674	$11,816,949
**Base Case**	**$0**	**−** **$1,415,057**	**$7,028,238**	**$9,089,833**	**$14,703,014**
Scenario Analysis *	**$0**	**$3,854,907**	**$3,938,368**	**$4,023,674**	**$11,816,949**

* Does not consider drug, administrative, and supportive costs. Values in the table above are subject to rounding.

**Table 11:** One-way sensitivity analysis results.

Parameter	Base Year: 2020	2021	2022	2023	3-Year Combined	% Change
Base Case	$0	−$1,415,057	$7,028,238	$9,089,833	$14,703,014	
**Adequate tissue for testing (84%)**						
74% (−10%)	$0	−$524,413	$8,409,393	$10,607,081	$18,492,061	26%
94% (+10%)	$0	−$2,305,700	$5,647,084	$7,572,584	$10,913,968	−26%
**Broad-based NGS testing performed (25%)**						
0% (−25%)	$0	$1,092,414	$11,703,143	$14,342,135	$27,137,692	85%
50% (+25%)	$0	−$3,922,527	$2,353,334	$3,837,530	$2,268,337	−85%
75% (+50%)	$0	−$6,429,997	−$2,321,571	−$1,414,773	−$10,166,341	−169%
100% (+75%)	$0	−$8,937,467	−$6,996,476	−$6,667,076	−$22,601,018	−254%
**Patients unable to continue due to tissue exhaustion (29%)**						
19% (−10%)	$0	−$3,970,100	$2,231,863	$3,697,009	$1,958,771	−87%
39% (+10%)	$0	$1,139,987	$11,824,614	$14,482,656	$27,447,258	87%

Values in the table above are subject to rounding.

## Data Availability

Medical cost data was obtained from the Ontario Case Costing Initiative. Clinical trial information was obtained from clinicaltrials.gov.

## Supplementary Materials

The following are available online at https://www.mdpi.com/article/10.3390/curroncol28060441/s1, Table S1: Drug Cost for 1-Year (without carry-over), Table S2: IV Drug Unit Costs, Table S3: Patient Characteristics, Table S4: Number of Cycles Calculations for IV Therapies, Table S5: Chemotherapy Treatment, Table S6: Chemo-immunotherapy Treatment, Table S7: Immunotherapy Treatment, Table S8: Immunotherapy Treatment, Table S9: Administrative Costs for Oral and IV treatments, Table S10: Supportive Costs for Oral and IV treatments.

## Appendix A

**Table A1:** Annual cost per patient per therapy.

Line of Therapy	Treatment	Drug Cost per Year	Administrative Cost per Year	Supportive Cost per Year	Cost Per Patient Per Therapy per Year
1L	Osimertinib *	$107,558.20	$194.33	$8192.96	$115,945.50
	Alectinib *	$123,224.00	$194.33	$8192.96	$131,611.30
	Crizotinib *	$94,900.00	$194.33	$8192.96	$103,287.30
	Entrectinib *	$104,386.35	$194.33	$8192.96	$112,773.65
	Carboplatin + Pemetrexed	$10,157.00	$5293.63	$2413.76	$17,864.38
	Pembrolizumab + Carboplatin + Pemetrexed	$133,307.14	$9722.99	$4433.43	$147,463.56
	Pembrolizumab	$131,212.19	$11,127.42	$5073.82	$147,413.43
2L	Carboplatin+ Pemetrexed	$10,157.00	$5293.63	$2413.76	$17,864.38
	Docetaxel	$4417.90	$2160.66	$985.21	$7563.77
	Nivolumab	$19,982.41	$2484.76	$1132.99	$23,600.17
	Pembrolizumab	$48,408.38	$4105.26	$1871.89	$54,385.54
	Atezolizumab	$27,465.39	$3024.93	$1379.29	$31,869.61

* These treatments are associated with carry-over as treatment duration spans over a year; 1L: first-line, 2L: second-line.

## Appendix B

**Table A2:** One-way sensitivity analysis results.

Parameter	Base Year: 2020	2021	2022	2023	3-Year Combined	% Change
Base Case	$0	−$1,415,057	$7,028,238	$9,089,833	$14,703,014	
**Lung cancer patients with NSCLC (85%)**						
75% (−10%)	$0	−$1,248,579	$6,201,387	$8,020,440	$12,973,248	−12%
95% (+10)	$0	−$1,581,534	$7,855,090	$10,159,225	$16,432,781	12%
**NSCLC patients with advanced disease (Stage 3B/4A) (63%)**						
53% (+10%)	$0	−$1,190,444	$5,912,645	$7,647,002	$12,369,203	−16%
73% (−10%)	$0	−$1,639,669	$8,143,832	$10,532,663	$17,036,826	16%
**Patients with non-squamous NSCLC (70%)**						
60% (−10%)	$0	−$1,212,906	$6,024,204	$7,791,285	$12,602,584	−14%
80% (+10%)	$0	−$1,617,207	$8,032,272	$10,388,380	$16,803,445	14%
**Patients who receive treatment (30%)**						
20% (−10%)	$0	−$943,371	$4,685,492	$6,059,888	$9,802,010	−33%
40% (+10%)	$0	−$1,886,742	$9,370,985	$12,119,777	$19,604,019	33%
**Biomarker not tested, excluding PD-L1 (28%)**						
18% (−10%)	$0	−$2,080,995	$6,943,984	$9,138,068	$14,001,057	−5%
38% (+10%)	$0	−$749,118	$7,112,493	$9,041,597	$15,404,972	5%
**Patients with insufficient tissue for biomarker testing (10%)**						
5% (−5%)	$0	−$1,888,215	$5,959,356	$7,865,877	$11,937,019	−19%
15% (+5%)	$0	−$941,898	$8,097,120	$10,313,788	$17,469,010	19%
**Adequate tissue for testing (84%)**						
74% (−10%)	$0	−$524,413	$8,409,393	$10,607,081	$18,492,061	26%
94% (+10%)	$0	−$2,305,700	$5,647,084	$7,572,584	$10,913,968	−26%
**Repeat biopsy performed (80%)**						
70% (−10%)	$0	−$1,030,780	$7,797,816	$9,961,022	$16,728,058	14%
90% (+10%)	$0	−$1,799,333	$6,258,660	$8,218,643	$12,677,971	−14%
**Broad-based NGS testing performed (25%)**						
0% (−25%)	$0	$1,092,414	$11,703,143	$14,342,135	$27,137,692	85%
50% (+25%)	$0	−$3,922,527	$2,353,334	$3,837,530	$2,268,337	−85%
75% (+50%)	$0	−$6,429,997	−$2,321,571	−$1,414,773	−$10,166,341	−169%
100% (+75%)	$0	−$8,937,467	−$6,996,476	−$6,667,076	−$22,601,018	−254%
**Patients unable to continue due to tissue exhaustion (29%)**						
19% (−10%)	$0	−$3,970,100	$2,231,863	$3,697,009	$1,958,771	−87%
39% (+10%)	$0	$1,139,987	$11,824,614	$14,482,656	$27,447,258	87%
**LBx-CGP (FoundationOne Liquid CDx^®^) discount**						
Discount (25%)	$0	−$2,543,748	$5,875,110	$7,911,727	$11,243,088	−24%
Discount (50%)	$0	−$3,672,440	$4,721,981	$6,733,621	$7,783,162	−47%
Discount (75%)	$0	−$4,801,132	$3,568,853	$5,555,515	$4,323,236	−71%
**Single-gene testing discount**						
Discount (25%)	$0	−$1,372,610	$7,071,604	$9,134,138	$14,833,132	0.9%
Discount (50%)	$0	−$1,330,163	$7,114,970	$9,178,443	$14,963,249	1.8%
Discount (75%)	$0	−$1,287,716	$7,158,335	$9,222,747	$15,093,366	2.7%
**Re-biopsy discount**						
Discount (25%)	$0	−$1,321,865	$7,123,448	$9,187,104	$14,988,687	1.9%
Discount (50%)	$0	−$1,228,673	$7,218,657	$9,284,376	$15,274,360	3.9%
Discount (75%)	$0	−$1,135,481	$7,313,867	$9,381,648	$15,560,033	5.8%
**Broad-based NGS**						
Discount (25%)	$0	−$1,385,730	$7,058,200	$9,120,443	$14,792,913	0.6%
Discount (50%)	$0	−$1,356,403	$7,088,161	$9,151,054	$14,882,812	1.2%
Discount (75%)	$0	−$1,327,077	$7,118,123	$9,181,664	$14,972,710	1.8%
