# Supplementary material for: Health and Budget Impact of Liquid-Biopsy-Based Comprehensive Genomic Profile (CGP) Testing in Tissue-Limited Advanced Non-Small Cell Lung Cancer (aNSCLC) Patients
Source: Curr Oncol. 2021 Dec 11;28(6):5278–94. doi: 10.3390/curroncol28060441 (PMC8700634; doi:10.3390/curroncol28060441)
Supplement: Supplementary file 1 [file curroncol-28-00441-s001.zip › curroncol-1493117-supplementary.pdf]

Current Oncology MDPI

Drug Costs

Patients are assumed to receive treatments in the reference or new scenario based on the products' monographs and approved treatment regimens used in Canada. The overall drug cost per patient was calculated based on the number of cycle in a year using the formula below. Table A1 represents the drug cost per treatment in a given year, without accounting for carry-over.

$$Overall\ Cost\ Per\ Patient = \left(\frac{cost}{mg} \times Recommended\ dose\ per\ cycle\right) \times number\ of\ cycles$$

Table S1. Drug Cost for 1-Year (without carry-over).

	Recommended	Description	Cycle Length (days)	Recommended dose per cycle (mg)	Unit cost	Cost/ mg	Number of cycles	Overall cost per patient
1L	Carboplatin +Pemetrexed	IV	21	Refer to IV Drug Cost Calculations			7.1	$10,157.00
	Pembrolizumab+ Carboplatin+ Pemetrexed	IV	21	Refer to IV Drug Cost Calculations			13.0	$133,307.14
	Pembrolizumab	IV	21	Refer to IV Drug Cost Calculations			14.9	$131,212.19
2L	Docetaxel	IV	21	Refer to IV Drug Cost Calculations			5.0	$4,417.90
	Nivolumab	IV	14	Refer to IV Drug Cost Calculations			5.5	$19,982.41
	Pembrolizumab	IV	21	Refer to IV Drug Cost Calculations			4.1	$48,408.38
	Atezolizumab	IV	21	Refer to IV Drug Cost Calculations			5.0	$27,465.39
**Targeted Therapies**								
1L	Osimertinib*	Oral-Tablet	1	80	80	$294.68	$3.68	365.0
	Alectinib*	Oral- Tablet	0.5	600	150	$42.20	$0.28	730.0
	Crizotinib*	Oral-Tablet	0.5	250	250	$130.00	$0.52	730.0
	Entrectinib*	Oral-Tablet	1	600	200	$95.33	$0.48	365.0

*These treatments are associated with carry-over; 1L- first-line, 2L- Second-line.

IV Drug Cost Calculations

Table S2. IV Drug Unit Costs.

Molecule	Cost	Strength in vial (mg)	Cost per mg
Pemetrexed	-	-	$0.62
Cisplatin	$135.00	50	$2.70
	$270.00	100	$2.70
Carboplatin	-	-	$1.29
Docetaxel	-	-	$11.56
Pembrolizumab	-	-	$44.00
Nivolumab	$782.22	40	$19.56
	$1,955.56	100	$19.56
Atezolizumab	$6,776.00	1200	$5.65

Note: Atezolizumab list prices 2019. Hoffmann-La Roche Data on file. All other drug costs were taken from DeltaPA.

Table S3. Patient Characteristics.

Median Patient Weight	68.20
Median BSA (m2)	1.76
Median Glomerular Filtration Rate (mL/min)	99.92

Table S4. Number of Cycles Calculations for IV Therapies.

Therapy	Duration of Exposure (months)	Duration of Exposure (days)	Number of cycles
**First-line Therapies**			
Carboplatin+ Pemetrexed	4.9	149.0	7.1
Pembrolizumab+ Carboplatin+ Pemetrexed	9.0	273.6	13.0
Pembrolizumab	10.3	313.1	14.9
**Second-line Therapies**			
Docetaxel	2.0	60.8	2.9
Nivolumab*	2.3	69.9	5.0
Pembrolizumab	3.8	115.5	5.5
Atezolizumab	2.8	85.1	4.1

Note: The length of a cycle is 21 days except Nivolumab, 14 days. Duration of exposure (days) = Duration of exposure (months) * 30.4 days.

First Line Treatments

Table S5. Chemotherapy Treatment.

Product	Recommended Dosage	Dose Needed per Administration (mg)	Cost per mg	Cost of Dose per cycle	Number of cycles	Cost per patient	Total cost per patient
Pemetrexed	500 mg/m² IV q21d	880.00	$0.62	$545.60	7.1	$3,870.12	$10,157.00
Carboplatin	AUC 5 IV q21d	687.06	$1.29	$886.31	7.1	$6,286.87	

Table S6. Chemo-immunotherapy Treatment.

Product	Recommended Dosage	Dose Needed per Administration (mg)	Cost per mg	Cost of Dose per cycle	Number of cycles	Cost per patient	Total cost per patient
Pemetrexed	500 mg/m² IV q21d	880.00	$0.62	$545.60	13.0	$7,108.39	
Carboplatin	AUC 5 IV q21d	687.06	$1.29	$886.31	13.0	$11,547.32	$133,307.14
Pembrolizumab	200 mg q21d	200.00	$44.00	$8,800.00	13.0	$114,651.43	

Table S7. Immunotherapy Treatment.

Product	Recommended Dosage	Dose Needed per Administration (mg)	Cost per mg	Cost of Dose per cycle	Number of cycles	Cost per patient	Total cost per patient
Pembrolizumab	200 mg q21d	200.00	$44.00	$8,800.00	14.9	$131,212.19	$131,212.19

Second Line Treatments

Table S8. Immunotherapy Treatment.

Product	Recommended Dosage	Dose Needed per Administration (mg)	Cost per mg	Cost of Dose per cycle	Number of cycles	Cost per patient	Total cost per patient
Docetaxel	75 mg/m² IV q21d	132	$11.56	$1,525.92	2.9	$4,417.90	$4,417.90
Nivolumab	3 mg/kg IV q14d	204.6	$19.56	$4,001.06	5.0	$19,982.41	$19,982.41
Pembrolizumab	200 mg q21d	200.00	$44.00	$8,800.00	5.5	$48,408.38	$48,408.38
Atezolizumab	1200 mg IV q21d	1200	$5.65	$6,776.00	4.1	$27,465.39	$27,465.39

Medical Costs

Medical costs in this model include administrative costs and supportive costs.

Administrative Costs

Administrative costs include: chair time, pharmacist, clinician consultation, and pre-medication cost. For oral treatments, the cost is calculated on a weekly basis, and for IV treatments, the costs calculated per every 21 days. Refer to Table A1 for the cost breakdown.

Table S9. Administrative Costs for Oral and IV treatments.

Administrative costs	IV treatments- cost per 21 day cycle	Oral treatments- cost per week
Chair time	$633.01	$0.00
Pharmacist cost	$0.00	$1.36
Clinician consultation cost	$84.21	$2.37
Pre-medication cost	$29.07	$0.00
Total:	$746.28	$3.73

Supportive Costs

Supportive costs include: clinician consultation, lab testing, and imaging costs. For oral treatments, the cost is calculated on a weekly basis, and the for IV treatments the costs are per every 21 days (typical cycle length). Refer to Table A2 for the cost.

Table S10. Supportive Costs for Oral and IV treatments.

Supportive costs	IV treatments- cost per 21 day cycle	Oral treatments- cost per week
Clinician consultation cost	$38.05	$4.39
Lab testing	$49.64	$5.73
Imaging	$252.60	$147.14
Total	$340.29	$157.25
